# Integrative gene and isoform co-expression networks reveal regulatory rewiring in stress-related psychiatric disorders

**DOI:** 10.1016/j.isci.2025.113342

**Published:** 2025-08-13

**Authors:** Ghalia Rehawi, Jonas Hagenberg, BeCOME study group, Optima study group, Philipp G. Sämann, Lambert Moyon, Elisabeth Binder, Markus List, Annalisa Marsico, Janine Knauer-Arloth

**Affiliations:** 1 Max Planck Institute of Psychiatry, 80804 Munich, Germany; 2 Institute of Computational Biology, Helmholtz Zentrum München, 85764 Neuherberg, Germany; 3 Data Science in Systems Biology, TUM School of Life Sciences, Technical University of Munich, 85354 Freising, Germany; 4 Munich Data Science Institute, Technical University of Munich, 85748 Garching, Germany

**Keywords:** Genetics, Molecular biology, Neuroscience, Omics

## Abstract

Isoform-specific expression patterns have been linked to stress-related psychiatric disorders such as major depressive disorder (MDD). To further explore their involvement, we constructed co-expression networks using total gene expression (TE) and isoform ratio (IR) data from affected (*n* = 210, 81% with depressive symptoms) and unaffected (*n* = 95) individuals. Networks were validated using advanced graph generation methods. Our analysis revealed distinct differences in network topology and structure. Shared hubs exhibited unique co-regulatory patterns in each network, with key master hubs in the affected network showing association with psychiatric disorders. Gene Ontology enrichment highlighted condition-specific biological processes linked to each network’s master hubs. Notably, isoform-level data uncovered unique co-regulatory interactions and enrichments not observed at the gene level. This is the first study to show network-level differences of gene and isoform co-expression between affected and unaffected individuals of stress-related psychiatric disorders, emphasizing the importance of isoforms in understanding the molecular mechanisms of these conditions.

## Highlights

• Gene and isoform networks differ significantly between affected and unaffected individuals
• Key hub nodes in the affected network are linked to psychiatric disorders
• GO analysis shows distinct biological processes in affected versus unaffected networks
• Isoform-level data reveal unique interactions and enrichments beyond gene-level analysis

## Graphical abstract

**Figure:**
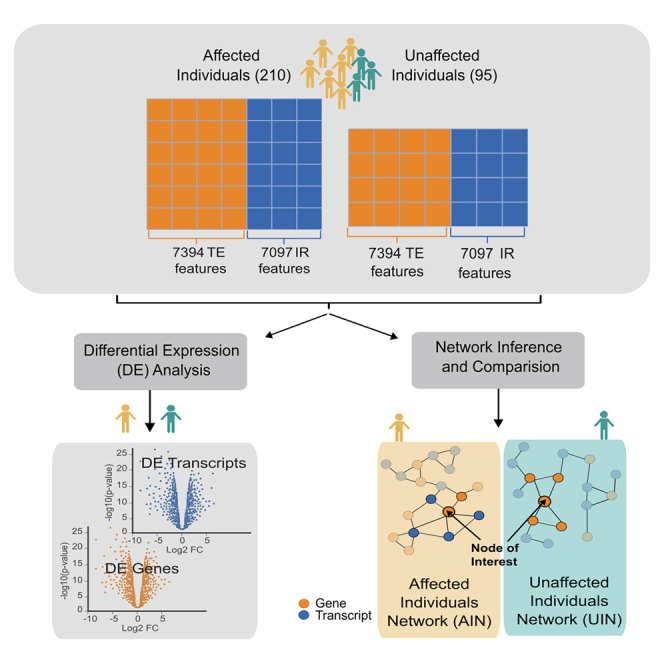

## Introduction

Stress-related psychiatric disorders, such as major depressive disorder (MDD), anxiety disorders, and post-traumatic stress disorders (PTSD) share common pathophysiological, clinical, and biological characteristics and impose a significant burden on individuals and society.^1^^,^^2^ These conditions disrupt thinking, mood, and daily functioning, leading to the diminished quality of life and often long-lasting disability. This burden extends to healthcare systems, where psychiatric disorders are a leading cause of disability and contribute to poor outcomes in physical diseases.^1^ MDD exemplifies the challenges in understanding and treating psychiatric disorders. As a highly polygenic disease, MDD is influenced by numerous genetic variants, and its high comorbidity with many other psychiatric disorders complicates its study.^3^ Cross-disorder psychiatric studies offer a valuable approach to investigating shared biological processes beyond phenotypic features.^4^

Through Genome-Wide Association Studies (GWASs), the majority of disease-associated variants were found to be in non-coding regions, highlighting the importance of gene expression and splicing regulation in contributing to genetic risk. This has led to increased interest in studying the gene expression landscape and transcriptional regulation. Differential expression analysis (DEA) is an important tool that allows researchers to identify genes expressed at significantly different levels between two or more conditions. Using samples from brain and blood tissues, many studies have identified transcriptional dysregulation patterns in patients with psychiatric disorders,^5^^,^^6^^,^^7^^,^^8^^,^^9^^,^^10^ with multiple differentially expressed genes being shared across several psychiatric disorders.^7^^,^^8^

To unravel the complex biology of psychiatric disorders, it is important to organize genes within their broader molecular system and pathway context. However, DEA often focuses on individual genes, potentially overlooking the complex interactions and regulatory relationships within biological systems. To this end, co-expression networks have emerged as a powerful tool. This approach involves constructing networks representing functional relationships between genes, where nodes represent genes and edges represent a correlation of expression patterns. Network methods allow researchers to identify key regulatory genes and modules of functionally related genes and link them to disease-related pathways, offering a more comprehensive view of the molecular mechanisms underlying psychiatric disorders.^11^^,^^12^^,^^13^

Network approaches have been widely used to investigate the pathophysiology of psychiatric disorders.^12^^,^^13^^,^^14^^,^^15^^,^^16^ For example, studies have used network methods to explore gene interactions, identifying key hub genes and modules associated with MDD status from blood samples.^14^^,^^16^ However, most existing methods have focused on investigating gene-level interactions,^8^^,^^12^^,^^13^^,^^16^ disregarding the effect of post/co-transcriptional modification processes, including alternative splicing (AS). AS affects up to 95% of human genes,^17^ plays an important role in gene regulation, and contributes to the diversity and complexity of the proteome^18^^,^^19^^,^^20^ by producing different isoforms of the same gene, with much research demonstrating that different isoforms of the same gene may have different or even opposing functions.^20^^,^^21^^,^^22^ Recently, considerable effort has been directed toward studying AS and splicing dysregulation in psychiatric disorders.^7^^,^^23^^,^^24^^,^^25^ For instance, an increase in the expression of specific isoforms of the neuregulin 1 receptor *ERBB4* has been reported in patients with schizophrenia.^26^ Another study identified differentially spliced genes, including splicing regulators, in patients with autism spectrum disorder (ASD).^27^ Studies incorporating isoform-level data into differential expression and network analysis have revealed larger effect sizes and more informative disease-specific transcriptional profiles and biological signals often missed when focusing solely on gene-level expression.^7^^,^^28^^,^^29^ For example, in a cross-disorder study of ASD, SCZ, and BP, Gandal et al.^7^ demonstrated that isoform-level co-expression networks were more strongly associated with disease-specific GWAS loci than gene-level networks.

While these studies have highlighted the importance of isoform-level analysis in understanding psychiatric disorders, there remains a need for integrative approaches that combine both gene-level and isoform-level data in a single network framework. To address this need, we introduce an integrative network approach to compare and unravel the complex underlying biology between a network of affected individuals (AIN) with stress-related psychiatric disorders (*n* = 210, 81% with depressive symptoms) and a network of unaffected individuals (UIN) (*n* = 95). As in the work studying tissue-specific transcription and splicing by Saha et al.,^30^ we combine both total gene expression values (TE) and isoform ratios (IR) as two node modalities in our networks. Using advanced graph generation and embedding techniques, we validate that these networks capture biologically meaningful distinctions between the two groups. We compare the two networks to reveal differences in co-regulatory patterns both at gene and isoform levels. Additionally, we prioritize key genes and isoforms within the AIN that may play pivotal roles in disease pathways and serve as potential targets for therapeutics.

To elucidate the advantages of our network-based approach over current standard methods such as differential expression analysis, we perform DEA at both gene and transcript levels, followed by pathway and GWAS enrichment analyses on both DEA results and network findings. By constructing and comparing integrative gene and isoform networks for affected and unaffected individuals, we reveal changes in regulatory relationships and gain insights that are not captured from differential expression analysis alone.

## Results

Using gene and transcript expression data from 305 individuals (210 affected and 95 unaffected by psychiatric disorders), we first performed standard single-gene differential expression analysis and subsequently compared these findings to those obtained from our integrative network-based approach.

### Differential expression analysis reveals distinct gene and transcript-level dysregulation

**Figure 1:**
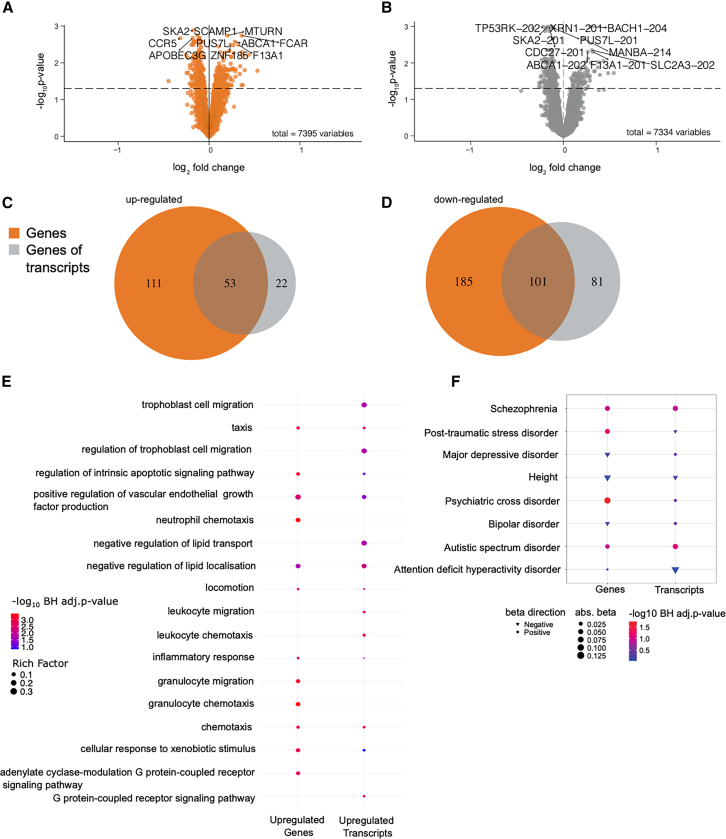
Results of differential gene expression (DGE, orange) and differential transcript expression (DTE, gray) analysis (A and B) Volcano plots visualize differentially expressed genes (A) and transcripts (B). See Tables S3, S4, and S7. The dashed line indicates the significance threshold of 5% FDR (*n* = 450 genes and 269 transcripts). The top 5 up- and down-regulated entities (based on FDR) are labeled. (C and D) Venn diagrams illustrate the overlap of upregulated (C) and downregulated (D) entities, see Figure S3. Orange represents genes, and gray represents genes of transcripts, where transcripts were mapped to their corresponding genes for overlap analysis. (C) Up-regulated: 53 genes found both at gene and transcript level, and 22 genes corresponding to 23 up-regulated transcripts found only at the transcript level. (D) Down-regulated: 101 genes found both at the gene and transcript level, and 81 genes corresponding to 81 down-regulated transcripts found only at the transcript level. (E) Significantly enriched (maximum of 10) Gene Ontology (GO) biological processes (based on BH adjusted *p*-values) are shown for upregulated genes and transcripts. Rich factor quantifying the degree of enrichment is indicated by the dot size, and the -log10 BH adjusted *p*-values are represented by the color code, ranging from blue (not significant) to red (significant). Non-significant enrichment values are also indicated by filled circles where available, otherwise left empty (no dots). (F) Enrichment of differentially expressed (DE) genes and transcripts in genes carrying SNPs associated with the GWAS traits schizophrenia, post-traumatic stress disorder, major depressive disorder, bipolar disorder, autistic spectrum disorder, attention deficit hyperactivity disorder, and a psychiatric cross-disorder GWAS. See Table S6. The GWAS trait of height was used as a baseline for comparison. Absolute beta values from the MAGMA analysis are indicated by the dot size, with negative beta values represented by a triangle and positive beta values by a circle. -log 10 of BH adjusted *p*-values are represented by the color code, ranging from blue (not significant) to red (significant).

After adjusting for biological variables (sex, age, BMI, and cell type composition), and technical variables (sequencing run, GC content, and total read pairs), we performed differential gene expression and differential transcript expression analyses incorporating both total gene expression counts (*n* = 7394 genes) and transcript expression counts (*n* = 7334 transcripts) from 229 affected and 107 unaffected individuals (see STAR Methods, Figure 1, and Table 1). Our DE analyses identified 450 differentially expressed genes (36% up-regulated) and 269 differentially expressed transcripts (30% up-regulated) at an FDR of 5% (Figures 1A and 1B; and Tables S3 and S4). Notably, we identified 104 transcripts showing differential transcript expression, while their parent genes did not show concurrent differential gene expression (Figures 1C and 1D; Figure S3). This indicates isoform-specific regulation, where the relative abundance of transcripts from these genes changes significantly despite stable overall gene expression, consistent with previous findings.^7^^,^^28^^,^^31^

**Table 1:** Cohort characteristics

Status	Study	N	Age	Female	BMI	Major depression or dysthymia diagnosis	BDI-II	Psychotropic drugs
Affected	all	229	39.6 (13.2)	59% (135)	25.3 (5.3)	81% (186)	24.9 (13)	47% (107)
	BeCOME	122	37.1 (12.5)	65% (79)	24.0 (4.4)	69% (84)	18.0 (12.8)	8% (10)
	OPTIMA	107	42.4 (13.5)	52% (56)	26.9 (5.8)	95% (102)	33.0 (7.7)	90% (97)
	IST	0	–	–	–	–	–	–
Unaffected	all	107	29.9 (9.1)	60% (65)	23.7 (3.8)	0% (0)	2.6 (5.2)	0% (0)
	BeCOME	70	32.5 (10.2)	66% (46)	23.4 (3.2)	0% (0)	4.0 (6.0)	0% (0)
	OPTIMA	0	–	–	–	–	–	–
	IST	37	25 (3.0)	51% (19)	24.4 (4.7)	0% (0)	4.0 (5.3)	0% (0)

This table presents cohort characteristics as percentages with absolute numbers in parentheses for categorical variables, or as means with standard deviations in parentheses for continuous variables. Depression diagnoses, including both full and subthreshold diagnoses in the last 12 months, were determined using the Munich-Composite International Diagnostic Interview (M-CIDI). Depression severity was assessed using the Beck Depression Inventory-II (BDI-II). The ,column psychotropic drugs refer to the percentage of subjects taking psychopharmacological medications during the study period. These medications include antidepressants, mood stabilizers, neuroleptics, tranquilizers, and herbal psychotropics.

To identify pathways and biological functions relevant to the differentially expressed genes and transcripts, we carried out enrichment analysis of differentially expressed genes and differentially expressed transcripts using ClusterProfiler,^32^ which revealed distinct biological processes associated with upregulated genes versus upregulated transcripts. Upregulated transcripts were uniquely enriched (Benjamini-Hochberg (BH) correction, *p* < 0.05) in leukocyte chemotaxis and leukocyte migration, both related to immune system processes. On the other hand, upregulated genes showed enrichment (BH, *p* < 0.05) in a different set of immune processes, including granulocyte chemotaxis, neutrophil chemotaxis, and granulocyte migration (Figure 1E; Table S5). Neither downregulated genes nor downregulated transcripts exhibited significant enrichment in any GO biological processes. To link our findings on differential gene and transcript expression to the genetic landscape of psychiatric disorders, we performed a GWAS enrichment analysis using MAGMA^33^^,^^34^ (Figure 1F). While the set of differentially expressed genes captured more cross-disorder-related genes (beta = 0.13, *p* = 0.04), the set of differentially expressed transcripts showed a larger effect size for enrichment in MDD-related SNPs (beta = 0.01, *p* = 0.70) compared to the set of differentially expressed genes (beta = −0.03, *p* = 0.70). Similarly, the set of differentially expressed transcripts showed a larger effect size for enrichment in bipolar disorder GWAS (beta = 0.01, *p* = 0.62) compared to the set of differentially expressed genes (beta = −0.01, *p* = 0.62) (see Figure 1F). The complete results of this analysis can be found in Table S6.

To identify the differentially expressed genes and transcripts most directly relevant to psychiatric disorders, we prioritized those with prior established associations in the literature. Specifically, we intersected all differentially expressed genes and transcripts (553 unique genes) with a self-curated list of genes known to be associated with psychiatric disorders (see Gene-disease association analysis in STAR Methods), yielding 53 genes, 15 of which are associated with depression (Table S7).

### Construction and validation of Co-expression networks

**Figure 2:**
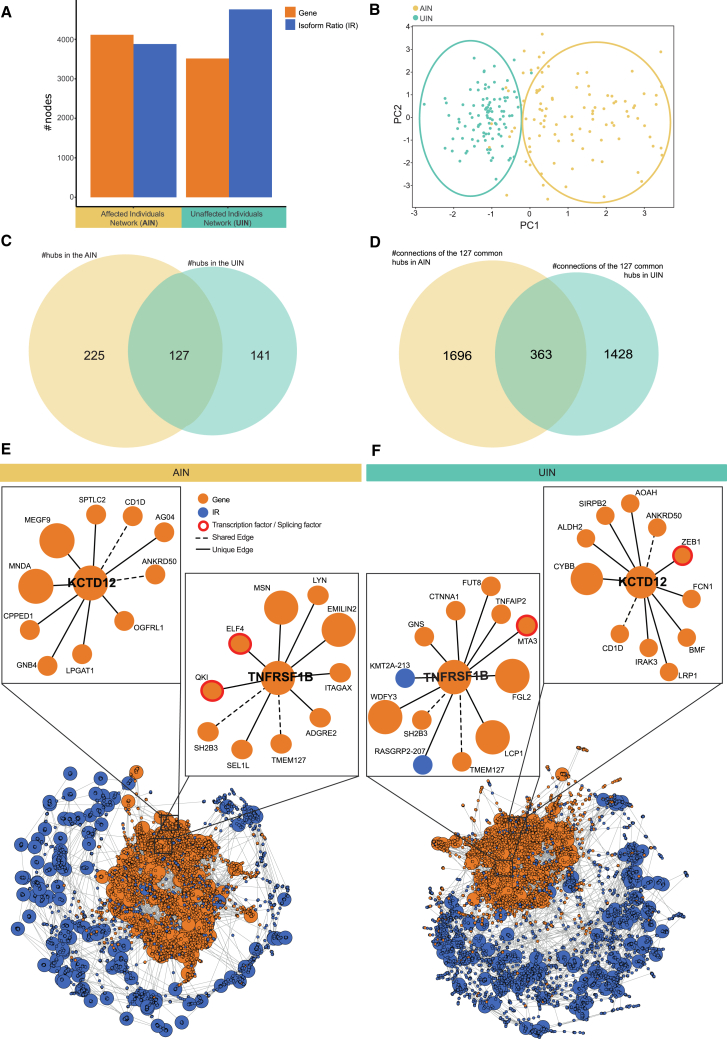
Results of network inference and investigation of common hubs (A) Barplot shows the number of total expression (TE, orange) and isoform ratio (IR, blue) nodes, separated by network: affected individuals (AIN, yellow) and unaffected individuals (UIN, green) after filtering and thresholding (AIN: 4115 TE nodes, 3881 IR nodes, 7996 total nodes; UIN: 3514 TE nodes, 4758 IR nodes, 8272 total nodes). (B) PCA plot of the Graph2vec embeddings of the simulated networks generated using the graph generative approach ARROWDiff. The PCA result shows a separate clustering of the 100 simulated UINs (green) and the 100 simulated AINs (yellow), validating the group-specific network topology and the robustness of network inference results. Dots indicate individual simulated networks. (C) Venn diagram shows the overlap of hub genes (degree ≥10) between AIN (Table S8) and UIN (Table S9), with 127 common hubs (Table S10). (D) Venn diagram illustrates the number of distinct connections of the 127 common hubs in the AIN and UIN, and their overlap. (E and F) Visualization of the full affected individuals’ network (AIN, e-bottom left) and unaffected individuals’ network (UIN, f-bottom right). Total gene expression of genes is represented by orange nodes. Isoform ratio (IR) nodes are represented by blue nodes. Zoomed-in views (top panels in E and F) focus on the first-order neighbors of two common hub genes associated with psychiatric disorders: *KCD12* and *TNFRSF1B*. Edges representing connections unique to either the AIN or UIN are represented by solid lines. Transcription and splicing factors are highlighted by red borders around the nodes.

To investigate differences in co-expression patterns of genes and isoforms between individuals affected (*n* = 210, 81% with depressive symptoms) and unaffected (*n* = 95) by stress-related psychiatric disorders, we constructed a co-expression network for each group and compared them. We used the information theoretic-based network inference approach ARACNE^35^ (see STAR Methods) using both corrected total gene expression (TE) values (*n* = 7394 genes) and isoform ratios (IR, *n* = 7097 ratios) (Figure S1A). To select only high-confidence edges in both networks, we applied thresholding based on the MI values (see STAR Methods). The resulting affected individuals’ network consists of 7996 nodes and 10,702 edges, while the network for unaffected individuals consists of 8272 nodes and 11,120 edges (see Table 2), with a similar number of TE and IR nodes (Figure 2A).

**Table 2:** Network statistics for the AIN and the UIN

Network	#Nodes	#Edges	#TE-TE edges	#TE-IR edges	#IR-IR edges
Affected-Individuals (AIN)	14,300	21,324	11951	1946	7427
	7996	10702	5997	983	3722
Unaffected-Individuals (UIN)	14,447	40,676	24199	6993	9484
	8272	11120	4837	1382	4901

This table provides an overview of the number of nodes and edges, as well as the number of edges for each edge type, in the affected individuals’ and unaffected individuals’ networks. Values are shown before (top row) and after (bottom row) the removal of edges with low mutual information (MI) values.

To assess whether the topological differences in our inferred networks are truly driven by underlying biological signals distinguishing the two groups, rather than being the result of random noise or limitations of the network inference method, we used graph generation and embedding techniques (see STAR Methods). Using the graph generation approach ARROW-Diff,^36^ we created 100 simulated graphs for each network. We then investigated the structural similarities between the generated graphs by embedding them using the Graph2Vec technique^37^ and then mapping the embeddings into a lower-dimensional representation using PCA (Figure S1B). The results revealed a clear separation between the two groups’ networks, with a classification accuracy of 0.92 using a logistic regression, Figure 2B). This quantitative assessment of the network topology distinctiveness between groups ensures robust inference and suggests that these networks reveal disease-specific patterns relevant to psychiatric diseases beyond noise.

### Differential network analysis reveals distinct co-expression patterns associated with stress-related psychiatric diseases

To pinpoint key differences between the networks of affected and unaffected individuals (illustrated in Figures 2E and 2F in the bottom), we focused on 1) investigating how common hub nodes differ in terms of connectivity patterns between the two networks, and 2) identifying key drivers of the underlying network-specific biology by investigating master hub nodes. To ensure that connectivity patterns are indeed distinct for each network, we only considered an edge to be distinct/unique to a network, if it is absent in the unthresholded version of the other network. By systematically comparing the connection patterns in the two networks, we aimed to uncover distinct patterns linked to psychiatric diseases both at the gene and isoform levels.

#### Common hub nodes show distinct connection patterns between the affected and unaffected individuals’ networks

Hub nodes, characterized by their high connectivity, are thought to play critical roles in the organization and regulation of biological networks, and alterations in their connectivity patterns can have significant functional consequences in disease states. To identify potential disruptions in these key regulatory elements in stress-related psychiatric disorders, we focused our analysis on hub nodes within the affected (AIN) and unaffected (UIN) individual networks (degree ≥10).

Hub nodes (degree ≥10) were identified in both networks. The AIN contained 352 hubs (Table S8) and the UIN 268 (Table S9), with 127 hubs common to both networks, as visualized in Figure 2C and detailed in Table S10). These common hub nodes showed a distinct connection pattern in each network. Specifically, in the AIN, 1,696 of all common hubs’ connections (82.4%) were unique. Similarly, 1,428 (79.7%) of all common hubs’ connections in the UIN were unique. Only 363 edges were shared between the two networks (Figure 2D).

To anchor this result, which suggests a rewiring pattern of common hubs, back to the context of psychiatric disorders, we searched the 127 common hubs (that include both TE and IR node types) for genes known to be implicated in psychiatric disorders. Of the 127 common hub nodes (Table S10), 22 genes and genes of corresponding transcripts showed associations with psychiatric disorders based on the DisGeNet resource (see STAR Methods).^38^ Six of which are differentially expressed at the gene level and three at the transcript level (Table S10). Figures 2E and 2F (e-bottom for AIN and f-bottom for UIN) illustrates the overall structure of the AIN und UNI, with zoom-in views (top panels in Figures 2E and 2F) depicting two of these 22 genes, the Potassium Channel Tetramerization Domain Containing 12 (*KCTD12*), which is associated with bipolar disorder,^39^ and the TNF Receptor Superfamily Member 1B (*TNFRSF1B*) associated with depression.^40^ These two genes exhibit substantially different connection patterns, with only 2 edges common to both networks for the two genes (dashed lines in the zoom-in view in Figures 2E and 2F).

#### Master hub nodes reveal genes and transcripts relevant to stress-related psychiatric disorders in the affected individuals’ network

**Figure 3:**
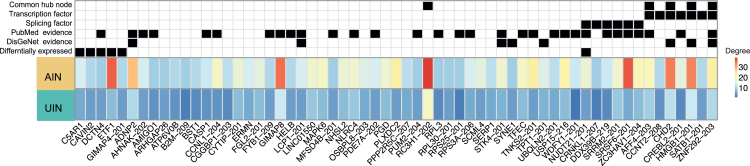
Master hub nodes in the AIN Heatmap of the 61 master hub nodes in the AIN (yellow) exhibiting a greater than 2-fold increase in degree compared to the unthresholded UIN (green). Node degree is indicated by a color gradient from blue (low) to red (high). The 61 master hubs are also annotated for different key information including DE status, roles as TFs or SFs, whether the master hub is also a common hub (degree ≥10 in both networks), and the existence of evidence associating each master hub to psychiatric disorders based either on DisGeNet or on our manual PubMed search (Table S11).

Our analysis revealed 61 master hub nodes in the AIN exhibiting substantial degree shifts, characterized by a minimum absolute fold increase of two in connectivity compared to the UIN, and a degree of at least 10. Of these, over half (*n* = 36) were IR nodes, with six also showing differential expression, including Complement C5a Receptor 1 (*C5AR1*), Caveolae Associated Protein 2 (*CAVIN2*), Dynactin Subunit 4 (*DCTN4*), Eukaryotic Translation Termination Factor 1 (*ETF1*), *GIMAP4-201* transcript of the gene GTPase, IMAP Family Member 4, and *NUDT21-201* transcript of the gene Nudix Hydrolase 21 (*NUDT21*) (Figure 3). The IR node *NUDT21-201* appears as a master hub of degree 17 in the affected individual’s network and is additionally a DE transcript (BH, *p* = 0.002, logFC = −0.11). The corresponding *NUDT21* gene is a known splicing factor and was identified in a recent study as a differentially expressed RNA-modification-related gene in MDD.^41^ In Table S11, we provide a list of all 61 master hub nodes in the AIN, with 31 (50%) showing evidence for association with psychiatric disorders based on a manual search in the PubMed repository, 17 of which appear in our analysis at the transcript level as IR master hubs. To summarize the results from this analysis of master hubs in the AIN and to draw better conclusions on potential new candidates for psychiatric disorders, we assembled all relevant information in Figure 3. The Figure shows the 61 master hub nodes of the AIN annotated for different key information including DE status, roles as TFs or SFs, whether the master hub is also a common hub (degree ≥10 in both networks), and the existence of evidence associating each master hub to psychiatric disorders based either on DisGeNet (see STAR Methods) or on our manual PubMed search (Table S11).

**Figure 4:**
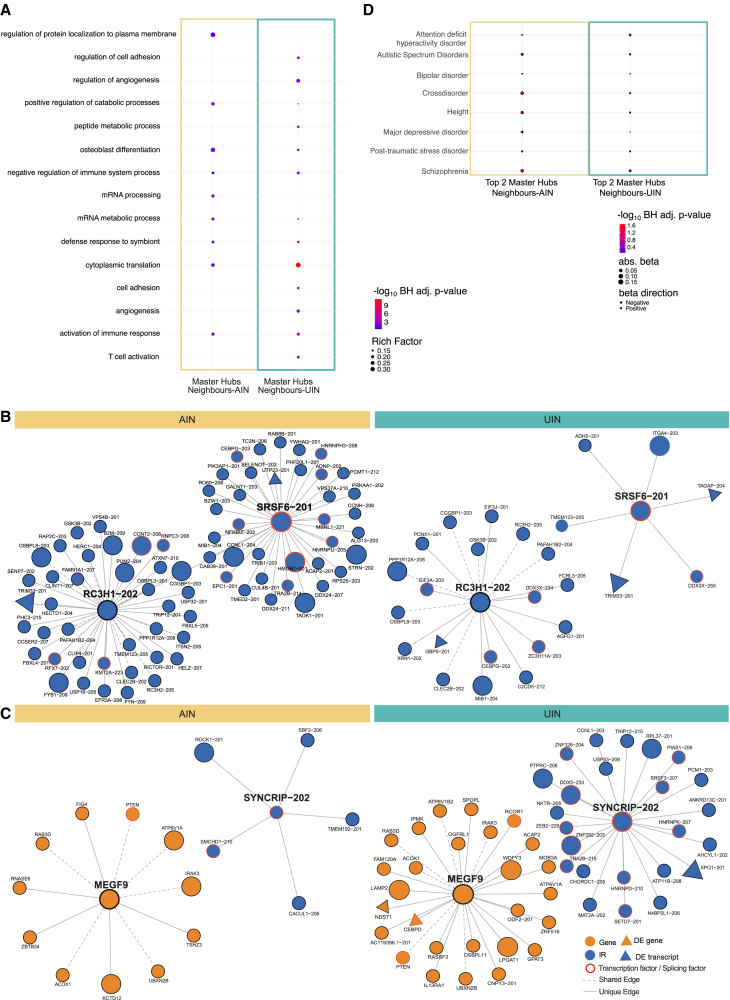
Combined analysis of master hubs in the AIN and the UIN (A) Significantly enriched (maximum of 10) GO biological processes (BH adj *p*-values) for the 61 master hubs and their first-order neighbors in the AIN and UIN. See Table S12. (B and C) Visualization of the Top 2 master hubs in each network. Total gene expression of genes is represented by orange nodes. Isoform ratio (IR) nodes are represented by blue nodes. Transcription and splicing factors are highlighted by red borders around the nodes. Edges representing connections unique to either the AIN or UIN are represented by solid lines. Network nodes that are differentially expressed in our DE analysis are represented by a triangle shape. (B) Top 2 master hubs (highest degree) in the AIN *RC3H1-202* and *SRSF6-201* (left) showing a distinct connection pattern compared to their corresponding nodes in the UIN (right). (C) Top 2 master hubs (highest degree) in the UIN *MEGF9* and *SYNCRIP-202* show distinct connection patterns compared to their corresponding nodes in the AIN (left). (D) Enrichment of the top 2 master hubs and their two-hop neighbors in each network in genes that carry SNPs associated with the GWAS traits schizophrenia, post-traumatic stress disorder, major depressive disorder, bipolar disorder, autistic spectrum disorder, attention deficit hyperactivity disorder, and a psychiatric cross-disorder GWAS. See Table S13. The GWAS trait of height was used as a baseline for comparison. Absolute beta values from the MAGMA analysis are indicated by the dot size, with negative beta values represented by a triangle and positive beta values by a circle. -log10 of BH adjusted *p*-values are represented by the color code, ranging from blue (not significant) to red (significant).

Similarly, we found 61 master hubs in the UIN showing twice as much connectivity compared to the AIN. A GO enrichment analysis of each network’s master hubs (61 each) and their first-order neighbors (AIN:1080, UIN:827), showed distinct biological processes related to each network (Figure 4A; Table S12). The set of master hubs of the UIN and their first-order neighbors indicated enrichments in the cytoplasmic translation, the activation of immune response, and cell adhesion processes (BH, *p* < 0.05), while the set of master hubs of the AIN and their first-order neighbors showed enrichment in other biological processes, including the positive regulation of catabolic process, osteoblast differentiation, and mRNA processing.

To further investigate whether master hubs reflect network-specific biology, we focused on the top two master hubs with the highest degree in each network. Figure 4B illustrates the top two master hubs in the AIN, *RC3H1-202* transcript of the gene Ring Finger And CCCH-Type Domains 1, and *SRSF6-201* transcript of the gene Serine and Arginine Rich Splicing Factor 6, along with their corresponding counterparts in the UIN. Similarly, Figure 4C presents the top two master hubs in the UIN, the Multiple EGF Like Domains 9 gene (*MEGF9*), and *SYNCRIP-202* transcript of the gene Synaptotagmin Binding Cytoplasmic RNA Interacting Protein (*SYNCRIP*), along with their respective versions in the AIN. In Figures 4B and 4C, we annotate the four master hubs and their neighbors with key information such as differential expression status and known roles as transcription or splicing factors. Focusing on these top two master hubs and their two-hop neighbors, we conducted a GWAS enrichment analysis for each network. Specifically, we investigated enrichment in genes harboring SNPs associated with psychiatric disorders (see STAR Methods). Our analysis revealed significant enrichment of *RC3H1-202*, *SRSF6-201*, and their two-hop neighbors (328 genes) in the AIN for genes implicated in cross disorder and schizophrenia (beta = 0.18, *p* = 0.03, beta = 0.16, *p* = 0.02 respectively). No corresponding significant enrichment was found using the set of genes comprising the master hubs *MEGF9*, *SYNCRIP-202*, and their two-hop neighbors (259 genes) in the UIN, see Figure 4D and Table S13.

#### Isoform ratio nodes show distinct co-regulatory patterns compared to their total expression nodes and capture different protein-protein interactions

**Figure 5:**
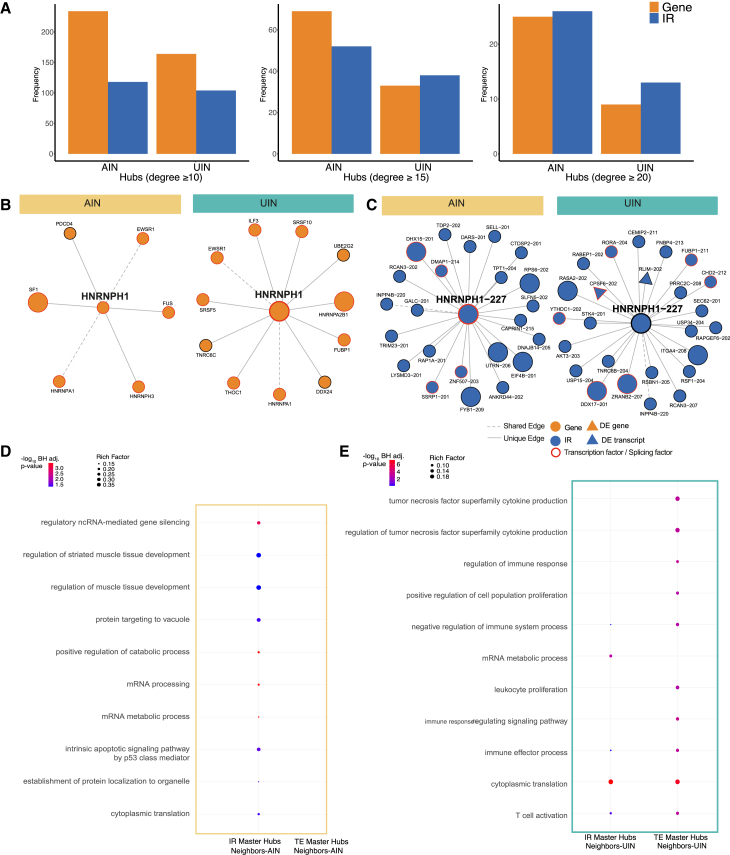
Isoform-specific network differences (A) Bar plots show the distribution of Isoform Ratio (IR) and Total Expression (TE) nodes in hubs with varying degree thresholds (≥10, ≥15, ≥20) in both the affected individuals’ network (AIN) and the unaffected individuals’ network (UIN). (B and C) *HNRNPH1* and *HNRNPH1-227* network visualizations: Total gene expression of genes is represented by orange nodes. Isoform ratio (IR) nodes are represented by blue nodes. Transcription and splicing factors are highlighted by red borders around the nodes. Edges represent connections unique to either the AIN or UIN are represented by solid lines. Network nodes that are differentially expressed in our DE analysis are represented by a triangle shape. (B) Network visualization of the heterogeneous nuclear ribonucleoprotein H1 (*HNRNPH1)* shows a distinct connection pattern in each network. *HNRNPH1* is an RNA-binding protein involved in pre-mRNA splicing and RNA processing and is known to be associated with neurodevelopmental disorders. (C) Network visualization of the hub IR node *HNRNPH1-227*, a transcript of the gene *HNRNPH1*, and its first-order neighbors. In both networks, first-order neighbors of *HNRNPH1-227* are substantially different from the first-order neighbors of its TE node *HNRNPH1*. (D and E) Significantly enriched (maximum of 10) GO biological processes (BH adj *p*-values) for master IR nodes and master TE nodes separately and for each network. Non-significant enrichment values are also indicated by filled circles where available, otherwise left empty (no dots). (D) In the AIN, IR master hubs and their first-order neighbors (36 IR master hubs and 627 neighbors) showed significant enrichment in GO biological processes, including in mRNA processing, mRNA metabolic process, positive regulation of catabolic process, and regulatory ncRNA-mediated gene silencing. No significant enrichments were found at the gene-level (TE master hubs and their neighbors). See Table S14. (E) In the UIN, considering IR master hubs and their first-order neighbors (30 IR master hubs and 413 neighbors) showed additional enrichment not found at the gene level, namely in mRNA metabolic processes. See Table S15.

Our analysis found that hub nodes are enriched for IR nodes, with the proportion of IR nodes exhibiting a positive correlation with increasing hub degree (e.g., ≥15 and ≥20, see Figure 5A). This observation underscores the critical role of IR nodes within these networks, prompting us to further investigate the unique contribution of isoform-level data by carrying out the following analysis. For each of the AIN and the UIN, we extracted the subnetwork comprising only TE-TE interactions and the subnetwork comprising only IR-IR interactions, and investigated their overlap. In the AIN, out of all 5997 connections of the TE-TE subnetwork, 5897 (98%) were unique, and out of 3722 connections of the IR-IR subnetwork, 3597 (96%) were unique to that subnetwork. Similarly, in the UIN, out of all 4837 connections of the TE-TE subnetwork, 4739 (98%) were unique, and out of all 4901 connections of the IR-IR subnetwork, 4777 (97%) were unique. These results highlight the distinct co-expression patterns captured by isoform-level data compared to gene-level. As an example, we present *HNRNPH1-227*, a hub IR node (degree ≥10) in both networks and a known transcription and splicing factor associated with neurodevelopmental disorders.^42^^,^^43^ In both networks, this node exhibits distinct connection patterns compared to its corresponding TE gene node, the Heterogeneous Nuclear Ribonucleoprotein H1 (*HNRNPH1*). Figures 5B and 5C depict the discrepancy between the connections of the gene node *HNRNPH1* and isoform ratio node *HNRNPH1-227* in both networks, along with annotated nodes information. The investigation of this node in the BioGrid database^44^ showed that *HNRNPH1-227* establishes connections with a unique set of proteins diverging from those observed at the gene level. More specifically, within the AIN, *HNRNPH1-227* interacts with isoforms of the proteins RPS6 and EIF4B, whereas the corresponding TE gene node captures interactions with EWSR1, SF1, HNRNPH3, HNRNPA1, and FUS. Likewise, within the UIN, *HNRNPH1-227* interacts with isoforms of the proteins CHD2, and DDX17, while the corresponding TE gene node interacts with EWSR1, HNRNPA1, SRSF5, HNRNPA2B, and ILF3. The observed differences in interaction patterns between isoform-specific nodes and their gene-level counterparts highlight the potential for isoform-level data to reveal previously unrecognized molecular relationships and functional specificities. To systematically explore whether these unique isoform-level co-expression patterns translate to distinct functional enrichments, we repeated the GO-enrichment analysis (section master hub nodes reveal genes and transcripts relevant to stress-related psychiatric disorders in the affected individuals’ network) on master IR nodes and master TE nodes separately and for each network. The results showed that in the AIN, IR master hubs and their first-order neighbors (36 IR master hubs and 627 neighbors) were significantly enriched in ten GO biological processes including mRNA processing, mRNA metabolic process, positive regulation of catabolic process, and regulatory ncRNA-mediated gene silencing. No significant enrichments were found from gene-level nodes alone, i.e., by considering TE master hubs and their first-order neighbors (25 TE master hubs and 453 neighbors) (Figure 5D; Table S14). Similarly, in the UIN, IR master hubs and their direct neighbors (30 IR master hubs and 413 neighbors) showed additional enrichment not found at the gene level, namely in mRNA metabolic processes (Figure 5E; Table S15). This result emphasizes the role of isoform-level data in uncovering condition-specific processes that are not visible when considering gene-level data alone.

## Discussion

In this work, we present an attempt to integrate gene expression and isoform ratio data into a comprehensive network approach to investigate co-expression patterns in individuals with and without stress-related psychiatric disorders, with a focus on depressive symptoms. This integrative approach allowed us to capture a deeper understanding of the complex interplay between multiple genes and isoforms, rather than focusing on the traditional gene-level analysis, revealing significant differences in network architecture between the two groups of individuals. We identified a total of 450 differentially expressed (DE) genes and 269 DE transcripts, including 104 with isoform-specific DE without corresponding DE at the gene-level. Of all DE genes and transcripts (mapped to 553 unique genes), we found that 53 genes have been shown to be associated with psychiatric disorders according to a DisGeNet query, 15 of which are associated with depression. Enrichment analyses on the DE genes and transcripts further highlighted the unique signal arising from isoform-level data to understand gene regulation in stress-related psychiatric disorders. Specifically, the set of upregulated transcripts showed significant enrichment in leukocyte chemotaxis, not captured from DE genes. Leukocytes in depressed patients show the dysregulation of genes involved in synaptic and neuroimmune functions, bridging the immune and the nervous system.^45^

While DE analysis has been widely used to study psychiatric disorders,^6^^,^^8^^,^^9^^,^^10^ incorporating isoform-level data into such analyses remains a relatively unexplored area. We compared our results to studies using blood samples from patients with MDD. Ota et al.^9^ found 8 DE genes in a longitudinal study of children and adolescents, with one gene overlapping with our DE genes: NADH:Ubiquinone Oxidoreductase Subunit A2 (*NDUFA2*). Wittenberg et al.^10^ conducted a meta-analysis of MDD across several blood transcriptome studies and provided a harmonized list of 272 DE genes. Of these, only six overlapped with our DE genes with concordant direction, namely *MBNL1* Antisense RNA 1 (*MBNL1-AS1*), IKAROS Family Zinc Finger 3 (*IKZF3*), and BUB3 mitotic checkpoint protein (*BUB3*) as down-regulated, and WD repeat domain 74 (*WDR74*), N-deacetylase and N-sulfotransferase 1 (*NDST1*), and Fc alpha receptor (*FCAR*) as up-regulated. Moreover, from the harmonized gene list, two genes, namely calcium/calmodulin-dependent protein kinase kinase 2 (*CAMKK2*), and protein tyrosine phosphatase non-receptor type 4 (*PTPN4*) appear in our analysis as DE solely at the transcript level (*CAMKK2-209*, and *PTPN4-201,* respectively) with no corresponding gene-level DE. Our differential transcript analysis and the unique enrichment profile of DE transcripts highlight the importance of isoform-level data, as it captures additional layers of transcriptional complexity that may be missed by traditional gene-level analyses.

While DE analysis provides information about individual genes and transcripts, it examines them in isolation, failing to capture the complex interplay between multiple genes and transcripts. Network co-expression analysis approaches represent a valuable tool, providing a comprehensive framework that captures the intricate interactions and functional relationships between genes and transcripts. Many studies have shown that the wiring of networks changes under different conditions, reflecting the pathophysiological states associated with diseases,^46^^,^^47^^,^^48^^,^^49^ such as in breast cancer.^48^ In psychiatric disorders, existing network approaches have focused on DE genes as input for network inference.^15^^,^^50^^,^^51^ However, hub nodes in biological networks have been found to play a critical biological function even if the gene itself does not show differential expression.^7^^,^^48^^,^^52^^,^^53^ Moreover, most methods focused on gene-level networks without considering isoform-level co-regulatory changes.^8^^,^^11^^,^^12^^,^^50^^,^^51^ While gene-level analyses provide valuable insights, incorporating isoform-level data is crucial for capturing the full complexity of gene regulation in psychiatric disorders. Isoforms can have distinct functions and interactions, and their dysregulation may contribute to disease pathogenesis in ways that are not apparent at the gene level.^24^^,^^25^^,^^54^

Therefore, we introduce an integrative approach combining both total gene expression values (TEs) and isoform ratios (IRs) to construct separate co-expression networks for affected and unaffected individuals (AIN vs. UIN), thereby using all genes and transcripts that passed our quality control (*n* = 7394, *n* = 7097). We ensured the robustness of ARACNE network inference in capturing biological differences between the two groups using advanced graph generation and embedding techniques. This marks the first attempt to leverage AI techniques, such as graph embeddings and graph generation, for the validation of network inference, enhancing the reliability of the network analysis results by confirming the distinctiveness of the network topologies. Hub nodes were a key focus of our network analysis. We identified hub nodes in both networks, with 127 common hubs showing distinct connection patterns between the AIN and UIN. Specifically, 82.4% of the edges connected to these common hubs in the AIN were unique to that network, while 79.7% of the edges connected to the same hubs in the UIN were unique to the UIN. This suggests a potential rewiring of regulatory interactions in psychiatric disorders, which could have downstream effects on pathways and cellular functions, such as synaptic plasticity, neurotransmitter signaling, or the immune response.^45^ Of the 127 common hubs, we found that 22 genes (and genes of transcripts) have known associations to psychiatric disorders according to a DisGeNet query. These 22 genes and their neighbors constitute important candidates for further investigation, which could provide insights into the molecular mechanisms underlying disease pathogenesis.

Focusing on the AIN, our analysis revealed 61 master hub nodes. Master hubs represent highly influential nodes, exhibiting at least 2-fold degree change in one network compared to the other. The investigation of these master hubs in the AIN showed significant associations with psychiatric disorders, particularly those related to psychiatric cross-disorder phenotype, suggesting their involvement in shared disease processes across different diagnoses. In a study by Wei et al.^51^ investigating MDD in the dentate gyrus (DG) and anterior cingulate cortex (ACC) regions of a mouse brain, the authors created an interaction network of DE genes and identified important differentially expressed TFs that regulate many hubs, including the TF gene chromodomain helicase DNA binding protein 2 (*CHD2*). *CHD2* was predicted to upregulate the expression of DE genes related to MDD in the DG region. In our analysis, *CHD2* appears as an important gene with differential expression found at the transcript level (*CHD2-201*). This transcript-level DE provides a more nuanced view of *CHD2* dysregulation compared to previous studies that focused on gene-level expression. Furthermore, *CHD2* is a master hub node in the AIN with a degree of 34 compared to 12 in the UIN, suggesting altered regulatory interactions in the context of psychiatric disorders. Further investigation of the 61 master hubs, especially those which are differentially expressed (*C5AR1, CAVIN2, DCTN4, ETF1, GIMAP4-201,* and *NUDT21-201*), may reveal novel therapeutic targets for psychiatric disorders, particularly depression, which represents the major portion of the clinical phenotype studied here.

To further investigate the functional roles of master hubs, we performed pathway enrichment analysis of the master hubs in each network and their first-order neighbors. The set of master hubs of the AIN and their first-order neighbors showed enrichment in the positive regulation of catabolic processes, osteoblast differentiation, and mRNA processes. The enrichment of mRNA processes in the AIN could suggest the dysregulation of RNA processing and splicing, potentially leading to altered isoform expression and downstream functional consequences. While seemingly unrelated to psychiatric disorders at first glance, enrichment in osteoblast differentiation may have intriguing implications. Osteoblast differentiation is crucial for bone formation and remodeling, and dysregulation of this process is implicated in osteoporosis. Studies have shown a complex interplay between osteoporosis, chronic stress, and inflammation.^55^ Chronic stress, a well-established risk factor for psychiatric disorders such as depression, activates the hypothalamic-pituitary-adrenal (HPA) axis, leading to prolonged cortisol release. Excess glucocorticoids suppress osteoblast differentiation by downregulating Wnt/β-catenin and insulin-like growth factor 1 (IGF-1) signaling, which is critical for bone formation.^56^ This suggests a potential link between the enriched osteoblast differentiation pathway in the AIN and the chronic stress often experienced by individuals with psychiatric disorders. Further investigation of this pathway may uncover novel connections between bone health, stress response, and mental health.

Since the UIN represents the healthy control group, pathways enrichments of the master hubs and their first-order neighbors may reflect normal biological processes and regulatory pathways that are essential for maintaining healthy functions in the body. For instance, the enrichment of cytoplasmic translation processes could be crucial for supporting protein synthesis and synaptic plasticity, which are essential for learning, memory, and cognitive flexibility. Since the list of significant biological processes associated with master hubs in the AIN and UIN is fully distinct, it suggests that the two networks represent different molecular pathways involved in disease pathogenesis, potentially leading to the identification of network-specific therapeutic targets.

Our analysis found that hubs and master hubs were enriched in isoform ratio nodes, underscoring the importance of isoform-level nodes in our networks. To further investigate the advantage of isoform-level data as another data modality, we extracted the subnetwork comprising all TE-TE interactions and the subnetwork comprising all IR-IR interactions. The results showed that, for each of the AIN and the UIN, the two subnetworks are largely distinct in their interactions (IR-IR interactions were mapped to their corresponding gene-level). These results highlight the distinct co-expression patterns captured by isoform-level data compared to gene-level data, and highlight the potential for isoform-level data to reveal previously unrecognized molecular relationships and functional specificities. Hence, to systematically explore the functional enrichments of isoform-level data, we carried out GO enrichment analysis on the TE master hubs and the IR master hubs separately and for each network. The results revealed that isoform-level data (IR master hubs and their first-order neighbors) are enriched in unique biological processes not captured at the gene-level. For example, in the AIN, the set of IR master hubs and their first-order neighbors was significantly enriched in ten GO biological processes including mRNA processing, mRNA metabolic process, and positive regulation of catabolic process, whereas no significant enrichment was found at the gene-level, i.e., by considering TE master hubs and their neighbors.

Throughout our analysis, we highlighted examples of hub and master hub nodes associated with psychiatric disorders, providing concrete evidence for the relevance of our network-based approach. Moreover, our study provides evidence for the importance of isoform-level analysis in understanding the complex landscape of gene regulation in psychiatric disorders. Consequently, our findings emphasize the need for the comprehensive functional annotation of isoforms to better understand their roles in complex biological processes and disease mechanisms.

### Limitations of the study

This work presents opportunities for future research to build upon our findings. For example, while we used ARACNE for network inference, which effectively captures non-linear relationships between genes and isoform ratios, future studies could assess the robustness of our findings using alternative approaches. Similarly, while our network analysis reveals co-regulatory changes and network-specific biomarkers, exploring alternative methods for differential network analysis such as BoostDiff^57^ and chNet,^48^ could provide an alternative way to understand network alterations. While module analysis represents a powerful approach for dissecting network organization, our initial attempts to identify condition-specific modules using spectral clustering were largely driven by the inherent differences between gene-level (TE) and isoform-level (IR) data (Figures 2E and 2F, bottom). This strong influence of data modality on module structure made it challenging to directly attribute module-level enrichments to the affected or unaffected states. However, we acknowledge the potential value of exploring network modules, particularly in the context of the distinct wiring patterns observed for common hub nodes. Future research could investigate alternative module detection algorithms or methods specifically designed for multi-modal network data to further elucidate the functional consequences of the observed regulatory rewiring in stress-related psychiatric disorders. Additionally, exploring gene expression patterns in other tissues, such as the central nervous system or the gut, could provide a more comprehensive understanding of the molecular processes underlying psychiatric disorders.^58^ A limitation of our study is the potential confounding effect of pharmacotherapy, given that a significant portion of the affected individuals were medicated. While we showed limited direct correlation between broad medication categories and key gene expression patterns, future research should prioritize more granular sensitivity analyses, including stratification by specific medications and dosages, as well as more sophisticated covariate adjustment techniques. Combining this with longitudinal data will be crucial for a more accurate understanding of disease mechanisms and their temporal dynamics, independent of treatment effects. The cell type heterogeneity in blood samples, especially the imbalance between PBMCs used for affected individuals and the mix of PBMCs and whole blood samples for unaffected individuals, could have affected our results. While we adjusted for different blood cell type compositions, further investigation using more homogenous cell populations or advanced deconvolution methods could refine our understanding of cell-type-specific effects. Finally, exploring alternative representations of splicing events, such as exon-level values, could offer more granular insights into splicing dynamics.

## Resource availability

### Lead contact

Requests for further information and resources should be directed to and will be fulfilled by the lead contact, Ghalia Rehawi (ghalia.rehawi@helmholtz-munich.de).

### Materials availability

This study did not generate new unique reagents.

### Data and code availability

• The raw and processed RNA-seq data have been deposited at GEO under accession numbers GSE289144 and GSE289146, and is publicly available as of the date of publication.
• All original code has been deposited at https://github.com/cellmapslab/NetIso/, and is available in this article’s supplemental information (Data S1), and is publicly available as of the date of publication.
• Any additional information required to reanalyze the data reported in this article is available from the lead contact upon request.

## Acknowledgments

We extend our gratitude to all participants of the BeCOME and OPTIMA studies, without whom our research would not be possible. Elisabeth Binder for her role in funding acquisition. Markus List’s contributions were supported by the 10.13039/501100007316Klaus Tschira Stiftung (KTS, Klaus Tschira Foundation, Grant 00.003.2024). Annalisa Marsico and Lambert Moyon acknowledge support by the 10.13039/501100002347BMBF Cluster4Future program CNATM. Dr. Knauer-Arloth’s contributions were supported by the 10.13039/100000874Brain & Behavior Research Foundation (NARSAD Young Investigator Grant, #28063). Ghalia Rehawi is supported by the 10.13039/501100001656Helmholtz Association under the joint research school “Munich School for Data Science – MUDS.”

## Author contributions

Ghalia Rehawi: writing - original draft, visualization, software, methodology, formal analysis, conceptualization. Jonas Hagenberg: software and writing - review and editing. Philipp Sämann: data curation and writing - review and editing. Lambert Moyon: writing - review and editing. Elisabeth Binder: funding acquisition and writing - review and editing. Annalisa Marsico: conceptualization, supervision, and writing - review and editing. Markus List: supervision and writing - review and editing. Janine Knauer-Arloth: writing - review and editing, visualization, supervision, conceptualization, and funding acquisition.

## Declaration of interests

The authors declare no conflict of interest.

## Declaration of generative AI and AI-assisted technologies in the writing process

During the preparation of this work the authors used ChatGPT in order to improve language and readability. After using this tool, the authors reviewed and edited the content as needed and take full responsibility for the content of the publication.

## STAR★Methods

### Key resources table

**Table:** 

REAGENT or RESOURCE	SOURCE	IDENTIFIER
**Biological samples**		

Human PBMC cells	BeCOME and OPTIMA cohorts	https://doi.org/10.1186/s12888-020-02541-z https://doi.org/10.1159/000535492
Human whole blood samples	IST cohort	https://doi.org/10.1159/000535492

**Deposited data**		

Raw and processed RNA-seq data from human PBMC cells	This paper	GEO: GSE289146
Raw and processed RNA-seq data from human whole blood samples	This paper	GEO: GSE289144
Code for data preprocessing, network inference, and all network-related analyses	This paper, GitHub	https://github.com/cellmapslab/NetIso/

**Software and algorithms**		

STAR aligner v2.7.7a	Dobin et al.	https://doi.org/10.1002/0471250953.bi1114s51
RSEM v1.3.3	Bo Li et al.	https://doi.org/10.1186/1471-2105-12-323
Cutadapt v2.10	Martin, M et al.	https://doi.org/10.14806/ej.17.1.200
Granulator v1.2.0	Bioconductor	https://doi.org/10.18129/B9.bioc.granulator
Limma v3.50.1	Bioconductor	https://doi.org/10.18129/B9.bioc.limma
ClusterProfiler v4.12.6	Bioconductor	https://doi.org/10.18129/B9.bioc.clusterProfiler
ARROW-Diff	GitHub	https://github.com/marsico-lab/arrow-diff
ARACNE	minet	
TFLink	Liska et al.^59^	Liska O, Bohár B, Hidas A, Korcsmáros T, Papp B, Fazekas D, Ari E (2022) TFLink: An integrated gateway to access transcription factor - target gene interactions for multiple speciesTFLink: An integrated gateway to access transcription factor - target gene interactions for multiple species. *Database*, baac083
BioRender	BioRender	https://www.biorender.com/

### Experimental model and study participant details

#### Samples selection

This study included 336 Caucasian participants selected based on the availability of matching RNA sequencing (RNAseq) and phenotypic data from three cohorts recruited at the Max Planck Institute of Psychiatry in Munich: The Biological Classification of Mental Disorders (BeCOME) study (ClinicalTrials.gov: NCT03984084,^60^), the Imaging Stress Test (IST) study, and The OPtimized Treatment Identification at the Max Planck Institute study (OPTIMA) (ClinicalTrials.gov: NCT03287362,^61^). Individuals were assessed as affected/unaffected based on the Munich-Composite International Diagnostic Interview (DIA-X/M-CIDI).^62^^,^^63^

In total, our samples comprised 229 affected individuals (BeCOME: 122, OPTIMA: 107) who met either threshold or subthreshold DSM-IV-based DIA-X/M-CIDI criteria for any substance use, affective or anxiety disorder, including post-traumatic stress disorder and obsessive-compulsive disorder, within the last 12 months of enrollment. 186 of these participants had a (subthreshold) DSM-IV diagnosis of major depression or dysthymia. Unaffected individuals (BeCOME: 70, IST: 37) were defined as those without any DSM-IV-based DIA-X/M-CIDI diagnosis. However, to focus on a more specific set of psychiatric disorders, cases with pure nicotine dependence (without any other comorbid diagnosis) were excluded from the affected group and moved to the unaffected group, resulting in a total of 107 unaffected individuals.

All participants were assessed by the Beck Depression Inventory (BDI) II^64^ and the Montgomery–Åsberg Depression Rating Scale (MADRS).^65^ An overview of the sample characteristics is provided in Table 1.

The studies were approved by the ethics board of the Ludwig Maximilians University (approval BeCOME:#350–14, OPTIMA:#17–395, IST:#121-14) and conducted in accordance with the Declaration of Helsinki.

### Method details

#### RNA extraction and sequencing

Blood samples were collected in the morning under fasted conditions. RNA was extracted from peripheral blood mononuclear cells (PBMCs) from OPTIMA and BeCOME cohorts and stored at the MPI biobank. Ribosomal RNA (rRNA) was depleted to enrich for messenger RNA (mRNA) and improve the detection of other transcripts using RiboCop rRNA Depletion Kits. Libraries were prepared with the Lexogen CORALL total RNA-Seq V1 Library Prep Kit and sequenced on a NovaSeq 6000 (Illumina, San Diego, USA) with a target depth of 30 million reads per sample, as previously described in more detail in.^66^ RNA was extracted from whole blood samples from the IST cohort. rRNA was depleted with RiboCop and libraries were prepared with the Lexogen CORALL total RNA-Seq V2 Library Prep Kit. Sequencing was performed on a NovaSeq 6000 in a separate batch with a target depth of 15.6 million reads per sample. Raw and processed sequencing data have been deposited in GEO under accession numbers GSE289144 and GSE289146.

#### RNA-seq alignment and QC

Paired-end FASTQ files were aligned against the GRCh38.p12 primary assembly using the GENCODE v31 annotation^67^ with STAR aligner v2.7.7a. Alignment was performed using the option quantMode = TranscriptomeSAM, following protocol 7 of Dobin and Gingeras,^68^ which involves generating a transcriptome index and using it for alignment and quantification to produce output in transcriptomic coordinates. Gene and transcript-level expression were then quantified using RSEM v1.3.3^69^ for paired-end reads.

Gene and transcript-level reads were filtered for unwanted sequences using Cutadapt^70^ v2.10. Zero-length reads were removed and only those with a count ≥ 10 in at least 95% of samples were retained, resulting in 9777 genes and 11427 transcripts.

Cell type deconvolution was calculated using Granulator v1.2.0^71^ and the LM22 reference matrix.^72^ Principal components (PCs) of the cell type proportions were calculated for inclusion in downstream models (see Methods S1).

To account for confounding effects from different sequencing runs, we first corrected the gene and transcript-level data for the sequencing run using the `removeBatchEffect` function from the limma R package.^73^^,^^74^ Subsequently, we performed Surrogate Variable Analysis (SVA)^75^ to identify additional hidden batch effects. A canonical correlation analysis (CCA) identified high correlations between the calculated SVs and the PCs of gene expression values (corrected for sequencing run), where e.g., sv1 was highly correlated with pc1 (r=0.99), and sv2 with pc2 (r=0.97), indicating that the svs effectively capture the variation represented by the pcs of gene expression data. The CCA also showed a high correlation between the GC content and pc1/sv1 (r=0.79), and a moderate correlation between the total read pairs and pc2/sv2 (r=0.46). We supported our CCA by ANOVA tests. Since these technical factors could confound our analysis, we further corrected gene and transcript-level data for GC content and total read pairs. We also removed the first five principal components of cell type proportions due to the different cell type composition across our samples, as well as the effect of sex, age, body mass index (BMI), as these biological factors could also introduce unwanted variation. Moreover, we investigated the effect of the different medications consumed by the affected individuals (Table 1) and found no significant association to the variation of gene expression data according to the ANOVA tests (Methods S1, Figure S2; Table S16). Finally, we removed genes and transcripts with negative values due to the subtraction of the modeled biological and technical effects, resulting in 7394 genes and 7334 transcripts, which we used for differential gene and transcript analysis.

We removed technical and biological covariates to ensure that network inference primarily reflects the intrinsic differences between the affected and unaffected groups, preventing the network structure from being influenced by unrelated variations. For consistency, we used the same batch-corrected data for both differential expression analysis and network analysis.

#### Differential gene expression and transcript expression analysis

We carried out differential gene expression (DGE) and differential transcript expression (DTE) analysis on the corrected data using the limma-trend method.^73^^,^^76^ For this purpose, we transformed the corrected gene and transcript count data to log2-counts per million values (logCPM) and fitted the linear models using the functions lmFit and eBayes from limma. The design matrix included diagnosis (affected/unaffected) as the main factor of interest. Significant genes and transcripts were identified at an FDR of 5%.

#### Isoform ratios

Changes in the expression values of isoforms are influenced by various splicing regulatory processes and were shown to be associated with many diseases.^26^^,^^77^^,^^78^^,^^79^ Depending on the study objective, isoform-level information can be represented and modeled in several ways, including absolute isoform counts, isoform proportions, or isoform ratios relative to their parent genes.^19^^,^^28^^,^^30^ Similar to the work of Saha et al.,^30^ we modeled isoform abundances as ratios. Isoform ratios normalize transcript expression to the overall gene expression, reducing biases from gene-level variability and highlighting isoform-specific changes. The isoform ratio (IR) for each transcript was computed by dividing the corrected and logCPM-transformed transcript counts by the corresponding gene counts. Mapping isoforms to their genes was done using GENCODE V31 annotation.^67^ We removed transcripts whose genes had been filtered at previous steps. When none of the isoforms of a gene were expressed (0/0 divisions), the mean of the IR across all samples was taken.^30^ Hence, the final dataset for network inference includes 7394 genes (TEs) and 7097 isoform ratios (IRs).

#### Regulatory network inference for affected and unaffected individuals

Before constructing the networks for the affected and unaffected individuals, we performed principal component analysis (PCA)^80^^,^^81^ on the logCPM gene count and the IR data separately. Outliers were identified based on a visual inspection of the first two principal components from the PCA, with a focus on data points that deviated by more than 2 standard deviations from the mean on either PC1 or PC2. A total of 31 outliers (19 affected and 12 unaffected) were removed. This resulted in 210 affected and 95 unaffected individuals being included in the network inference.

For both the affected individuals’ and unaffected individuals’ networks, we used ARACNE (Algorithm for the Reconstruction of Accurate Cellular Networks),^35^ an information theoretic-based method designed for the reverse engineering of regulatory networks. ARACNE uses mutual information (MI) to identify potential interactions and the data processing inequality (DPI) to remove indirect relationships.^82^ We used both gene expression and isoform ratios as input for ARACNE and removed edges connecting features of the same gene to reduce bias,^30^ see Methods S1 and Figure S1A.

The initial results showed that the UIN has twice as many edges compared to the AIN with lower MI values of the inferred edges. This is likely due to a reduced inference power caused by the smaller sample size in the unaffected group (see Methods S1 and Figure S4). To enable meaningful and fair comparison, we applied a threshold based on the median MI value of each edge type in the affected network (Figure S5), resulting in two networks with similar statistics (Table 2 and Methods S1).

#### Validation of topological differences between the constructed networks

In this analysis, we assessed the robustness of the network inference step in capturing group-specific biological processes reflected in a distinct topological structure, as schematically illustrated in Figure S1B. To this end, we leveraged ARROW-Diff,^36^ a novel approach for efficient large-scale graph generation. ARROW-Diff incorporates two key components in an iterative procedure to generate graphs that closely resemble an input ground truth graph in terms of structural properties. The first component is an auto-regressive random walk-based diffusion model, which learns the generative process of random walks sampled from the input ground truth graph. This model captures the original network’s structural characteristics and local connectivity patterns. The second component is a Graph Convolutional Network (GCN),^83^ which is trained to predict the validity of the proposed edges from the first component.

Utilizing ARROW-Diff, we generated 100 graphs for each network (affected and unaffected individuals’ networks). These simulated graphs capture the structure of the corresponding input graph in terms of various graph statistics, including global clustering coefficient, triangle count, assortativity, and other relevant graph metrics on which the graph generation is evaluated. By generating simulated networks that resemble each of the affected/unaffected individuals’ networks, we introduce variability/noise to the networks inferred by ARACNE. This is because ARROW-Diff can add or remove edges from the generated graph while maintaining the intrinsic graph structure.

We hypothesize that if the network inference captures group-specific properties reflected in distinct topological structures, the simulated graphs should also exhibit such distinctiveness between the two groups, even with the introduced variability. To analyze the structural similarities and differences between the generated graphs, we employed a two-step dimensionality reduction approach. First, we embedded the 200 generated graphs using Graph2Vec^37^ with an embedding dimension of 128 in order to capture the structure of such large graphs. This step transforms each graph into a high-dimensional vector representation, capturing its topological features. Subsequently, we applied a PCA^80^ to map these high-dimensional embeddings into a lower-dimensional space, facilitating visualization and analysis of the clustering patterns among the embedded graphs. To assess how well the Graph2Vec embeddings can predict a binary class label 1 vs. 0 (affected vs. unaffected), we fit a logistic regression model on the 100 simulated AINs and the 100 simulated UINs, we split the data into 80% for training and 20% for testing and report the mean accuracy representing the fraction of correct predictions.

#### Functional enrichment

##### Enrichment analysis of biological processes and psychiatric risk

We conducted an enrichment analysis of the DE genes, DE transcripts, and master hubs’ neighbors within the networks of affected and unaffected individuals using enrichGO from ClusterProfiler^32^ v4.12.6. This analysis was based on Gene Ontology (GO) biological processes.^84^^,^^85^ To enhance the clarity and interpretability of the results, we applied the simplification process from ClusterProfiler with default parameters to remove redundancy among enriched GO terms, focusing on the most representative biological processes. Furthermore, using the Generalized Gene-Set Analysis of GWAS Data (MAGMA),^33^^,^^34^ we assessed the enrichment in genes carrying single nucleotide polymorphisms (SNPs) with genome-wide association to the following traits: Attention Deficit Hyperactivity Disorder (ADHD), Autistic Spectrum Disorder (ASD), Bipolar Disorder (BP), Major Depressive Disorder (MDD), Post-traumatic Stress Disorder (PTSD), and a Psychiatric Cross Disorder phenotype. To provide a comparative baseline, we included a GWAS for Height as a control trait. To run MAGMA, we used the NCBI gene location file build 37 and the 1,000 Genomes reference data file for SNP locations.

##### Gene-disease association analysis for psychiatric disorders

To investigate the associations between genes and psychiatric disorders, we used the DisGeNET resource, a comprehensive platform integrating information on gene-disease associations from various expert-curated databases, GWAS catalogs, animal models, and scientific literature.^38^ We queried DisGeNET for a wide range of mental and psychiatric conditions, including anxiety disorders, major depressive disorder, and various substance abuse and dependency disorders (Table S1). To ensure the reliability of our analysis, we applied a filtering criterion, considering only gene-disease associations (GDAs) with a score greater than 0.4. We used gene names of both genes and genes of corresponding isoforms for all enrichment analyses according to the GENCODE V31 annotation.

#### Network annotation

We compiled an extensive list from multiple resources to annotate the nodes within the networks for known transcription and splicing regulators. For splicing factors, we integrated data from SpliceAid-F, a curated database of human splicing factors and their RNA binding sites,^86^ which provided 67 splicing factors. Additionally, we incorporated a collection of 277 genes involved in pre-mRNA splicing from,^87^ and 406 splicing factor genes from.^88^ For transcription factors, we leveraged the TFLink resource,^59^ a gateway for transcription factor-target gene interactions. This integration resulted in a final compilation of 1,606 known transcription factors and 517 known splicing factors (Table S2). To maintain consistency and facilitate cross-referencing, we utilized gene names for the annotation process throughout our analysis.

### Quantification and statistical analysis

ANOVA tests for the batch effects exploratory analysis (Methods S1) was performed using the built-in R functions anova and lm. Differential gene expression (DGE) and differential transcript expression (DTE) analysis were performed using the limma-trend method from the R package limma. For adjusting the *p*-values for multiple testing, we used the toptable function from the limma package, employing its default parameters, which applies the Benjamini-Hochberg (BH) method to control the False Discovery Rate (FDR). Significant genes and transcripts were identified at an FDR of 5%.

## Supplemental information

Document S1. Figures S1–S5 and Methods S1

Table S1. Gene-disease associations extracted from DisGeNET, related to STAR Methods

Table S2. A pool of known transcription and splicing factors from multiple resources, related to STAR Methods

Table S3. Differential gene expression analysis results, related to Figure 1

Table S4. Differential transcript expression analysis results, related to Figure 1

Table S5. GO-enrichment analysis results of upregulated genes and transcripts, related to STAR Methods

Table S6. MAGMA gene-set analysis of DE genes and DE transcripts, related to Figure 1

Table S7. Intersection set of DE genes and DE transcripts with genes associated to psychiatric disorders, related to Figure 1

Table S8. Hub nodes (dg => 10) of the affected individuals’ network, related to Figure 2

Table S9. Hub nodes (dg => 10) of the unaffected individuals’ network, related Figure 2

Table S10. Common hub nodes to the affected and unaffected individuals’ networks, related to Figure 2

Table S11. The 61 master hubs of the affected individuals’ network and their association to psychiatric disorders based on manual PubMed querying, related to Figure 3

Table S12. GO enrichment analysis results the 61 master hubs in each network and their first-order neighbors, related to Figure 4

Table S13. MAGMA gene-set analysis of the top two master hubs in each network and their two-hop neighbors, related to Figure 4

Table S14. GO enrichment analysis results of IR master hubs and TE master hubs (with their first-order neighbors) of the affected individuals’ network, related to Figure 5

Table S15. GO enrichment analysis results of IR master hubs and TE master hubs (with their first-order neighbors) of the unaffected individuals’ network, related to Figure 5

Table S16. ANOVA test results (-log10 p-values) between the different medication categories and the first 10 PCs of genes expression values, related to STAR Methods

Data S1. Original code
