# Supplementary material for: Integrative gene and isoform co-expression networks reveal regulatory rewiring in stress-related psychiatric disorders
Source: iScience. 2025 Aug 13;28(9):113342. doi: 10.1016/j.isci.2025.113342 (PMC12414837; doi:10.1016/j.isci.2025.113342)
Supplement: Document S1. Figures S1–S5 and Methods S1 [file mmc1.pdf]

iScience, Volume *28*

Supplemental information

Integrative gene and isoform co-expression networks reveal regulatory rewiring in stress-related psychiatric disorders

Ghalia Rehawi, Jonas Hagenberg, BeCOME study group, Optima study group, Philipp G. Sämann, Lambert Moyon, Elisabeth Binder, Markus List, Annalisa Marsico, and Janine Knauer-Arloth

Supplementary Information

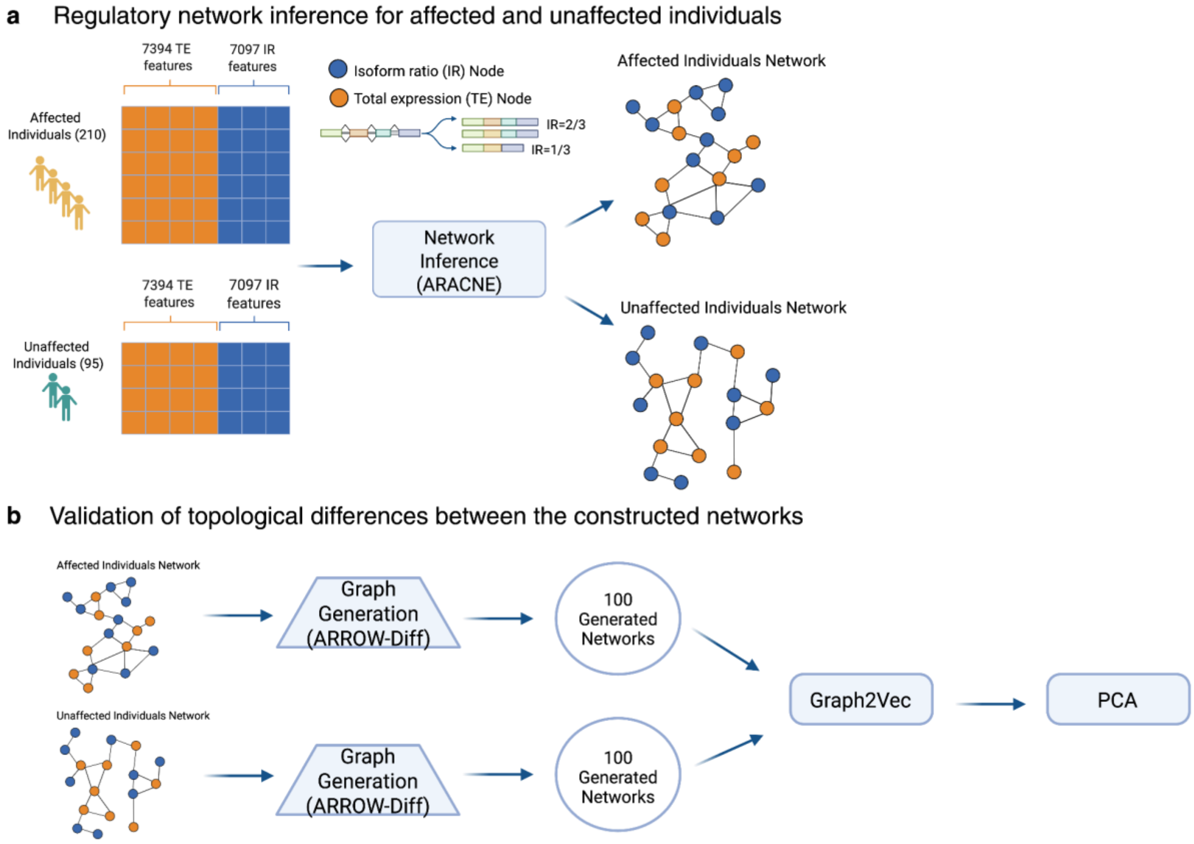

Figure S1: **Inference and validation of the AIN and the UIN.** Related to STAR Methods. (**a**) Networks for affected (210) and unaffected (95) individuals were built using ARACNE network inference by incorporating both total gene expression values (TEs) and isoform ratios (IRs). (**b**) We employed graph AI techniques, including the graph generation approach ARROW-Diff and the graph embedding approach Graph2Vec, to validate the distinct and network-specific topology for the inferred affected and unaffected individuals' networks.

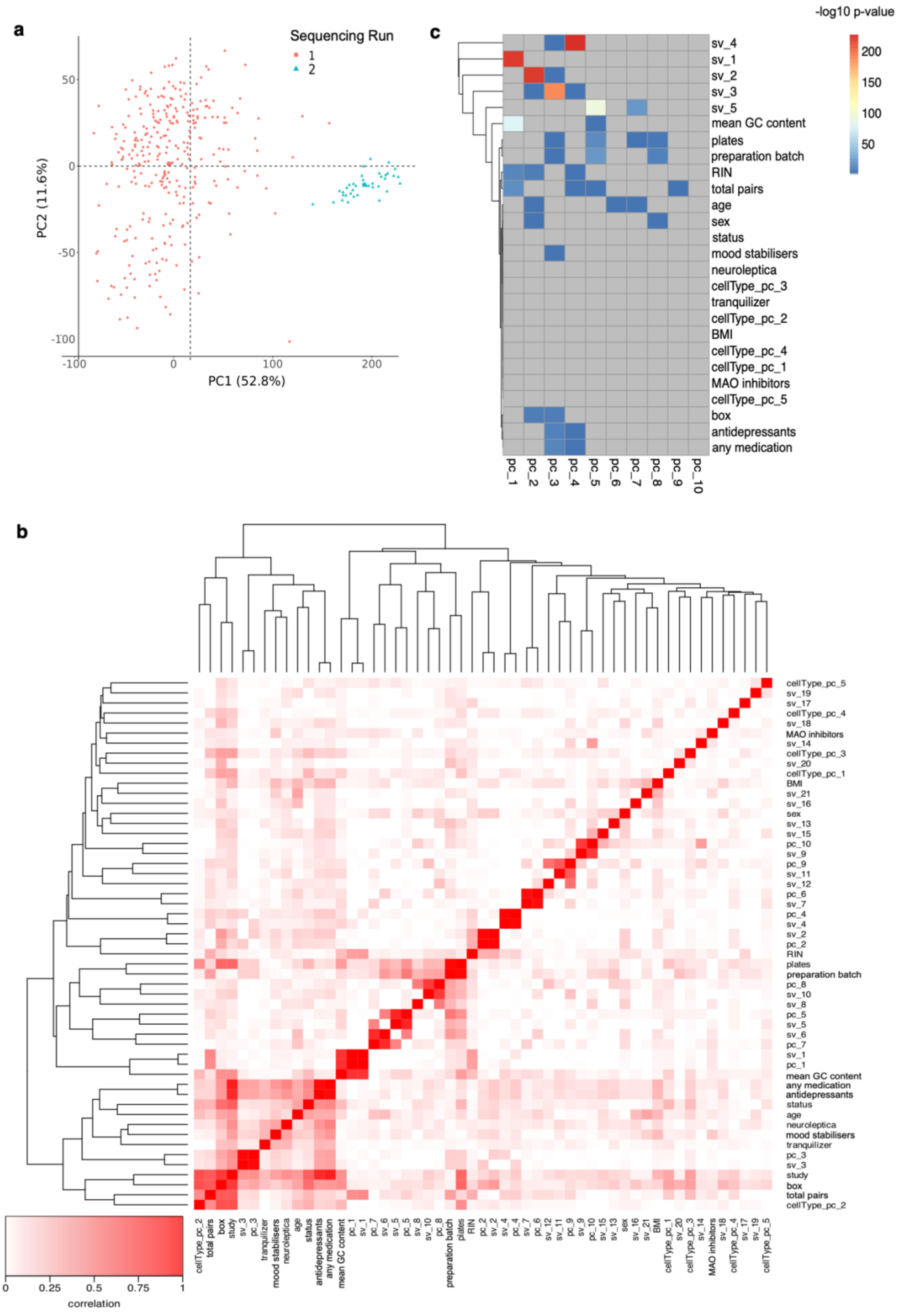
a
Sequencing Run
1
2
PC2 (11.6%)
PC1 (52.8%)
50
0
-50
-100
-100
0
100
200
c
-log10 p-value
200
150
100
50
sv_4
sv_1
sv_2
sv_3
sv_5
mean GC content
plates
preparation batch
RIN
total pairs
age
sex
status
mood stabilisers
neuroleptica
cellType_pc_3
tranquilizer
cellType_pc_2
BMI
cellType_pc_4
cellType_pc_1
MAO inhibitors
cellType_pc_5
box
antidepressants
any medication
pc_1
pc_2
pc_3
pc_4
pc_5
pc_6
pc_7
pc_8
pc_9
pc_10
b
cellType_pc_5
sv_19
sv_17
cellType_pc_4
sv_18
MAO inhibitors
sv_14
cellType_pc_3
sv_20
cellType_pc_1
BMI
sv_21
sv_16
sex
sv_13
sv_15
pc_10
sv_9
pc_9
sv_11
sv_12
pc_6
sv_7
pc_4
sv_4
sv_2
pc_2
RIN
plates
preparation batch
pc_8
sv_10
sv_8
pc_5
sv_5
sv_6
pc_7
sv_1
pc_1
mean GC content
any medication
antidepressants
status
age
neuroleptica
mood stabilisers
tranquilizer
pc_3
sv_3
study
box
total pairs
cellType_pc_2
0
0.25
0.5
0.75
1
correlation

Figure S2: **Batch effects analysis.** Related to STAR Methods. (**a**) The first and second principal components of a PCA show the separate distribution of samples based on the RNA-sequencing run, with (1) being the samples sequenced from OPTIMA and BecOME cohorts and (2) the samples sequenced separately from the IST study. (**b**) Heatmap showing the canonical correlations between all pairwise variables including technical and biological covariates, 21 SVs extracted after correcting for sequencing run, the first 5 PCs of cell type decomposition values, the first 10 PCs of the gene counts after correcting for sequencing run, and medication information split into the six categories: 'antidepressants', 'mood stabilisers', 'neuroleptica', 'tranquilizer', 'MAO inhibitors', and 'any medication'. (**c**) Heatmap of $-\log_{10}$ p values from ANOVA tests between known technical and biological covariates, 5 SVs extracted after correcting for sequencing run, the first 5 PCs of cell type decomposition values, the six categories of medication, and the first 10 PCs of the gene counts after correcting for sequencing run.

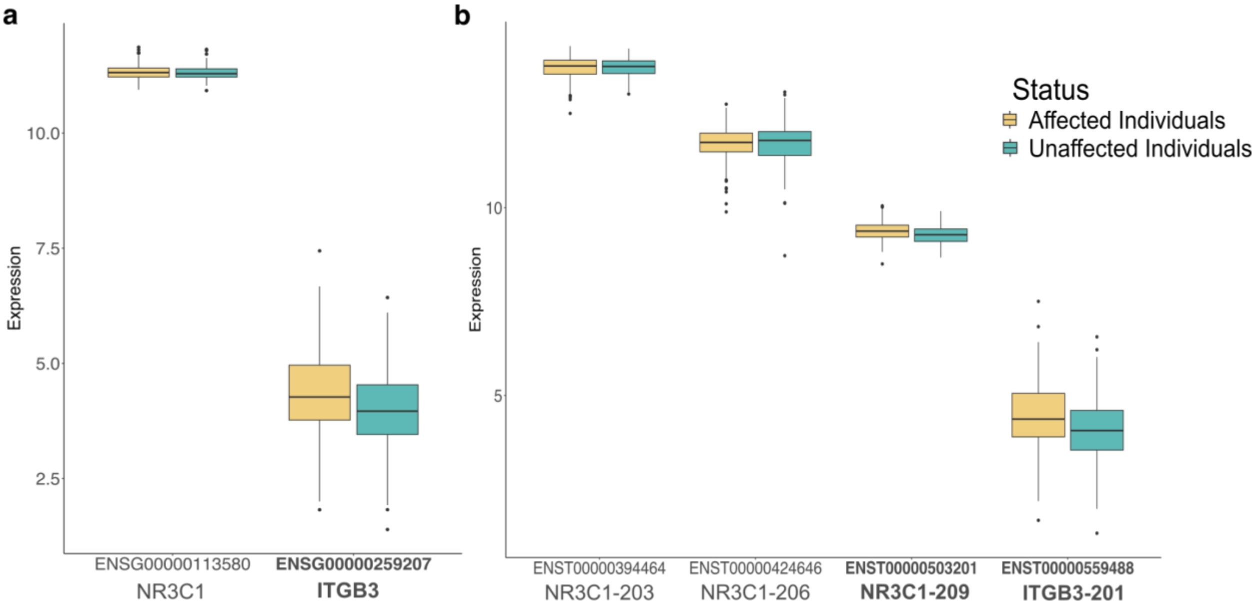

Figure S3: Gene-level vs. transcript-level expression of two genes *NR3C1* and *ITGB3*. Related to Figure 1c,d. (**a**) The expression in the affected and the unaffected groups of two genes, *ITGB3* (ENSG00000259207), which is up-regulated (bold), and *NR3C1* (ENSG00000113580), which is not differentially expressed. (**b**) The expression in the affected and the unaffected groups of 4 transcripts, 3 transcripts of the gene *NR3C1*, which does not show differential expression at the gene level, but one of the transcripts, *NR3C1-209* (ENST00000503201), is up-regulated (bold). The up-regulation of the gene *ITGB3* is also visible at its transcript level, where *ITGB3-201* (ENST00000559488) is also up-regulated (bold).

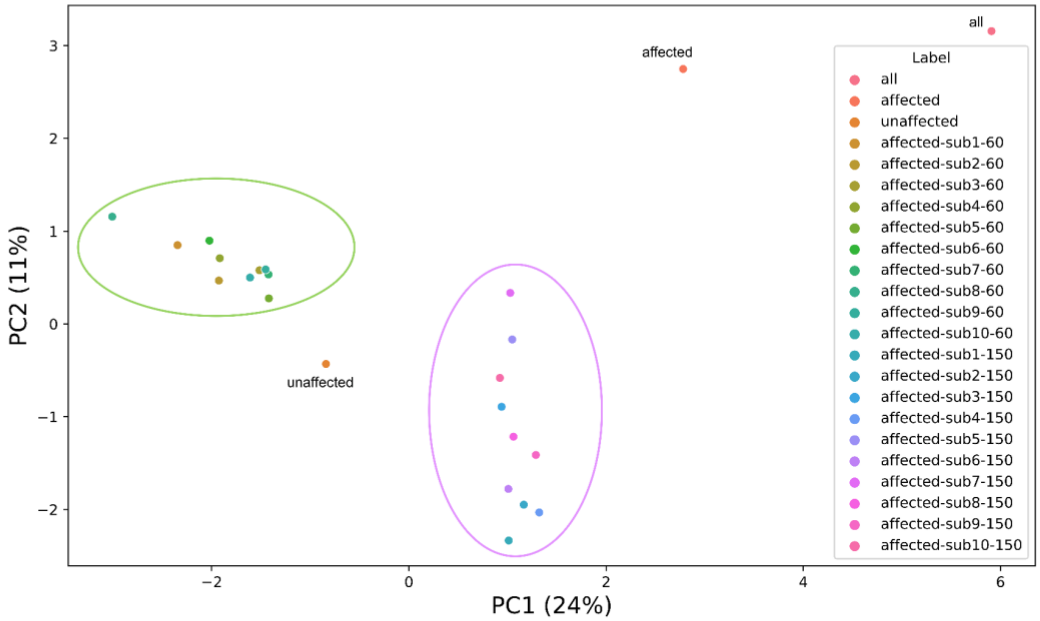

Figure S4: Sample size effect on network inference outcome. Related to STAR Methods. The first and second PCs of a PCA on graph embeddings obtained using Graph2Vec. The graph embeddings are of networks of different sizes: the full affected individuals' network (n=210), the full unaffected individuals' network (n=63), a combined network for affected and unaffected individuals (all), 10 sub-networks built using 60 samples from the affected group (green ellipse), and 10 sub-networks built using 150 samples from the affected group (violet ellipse).

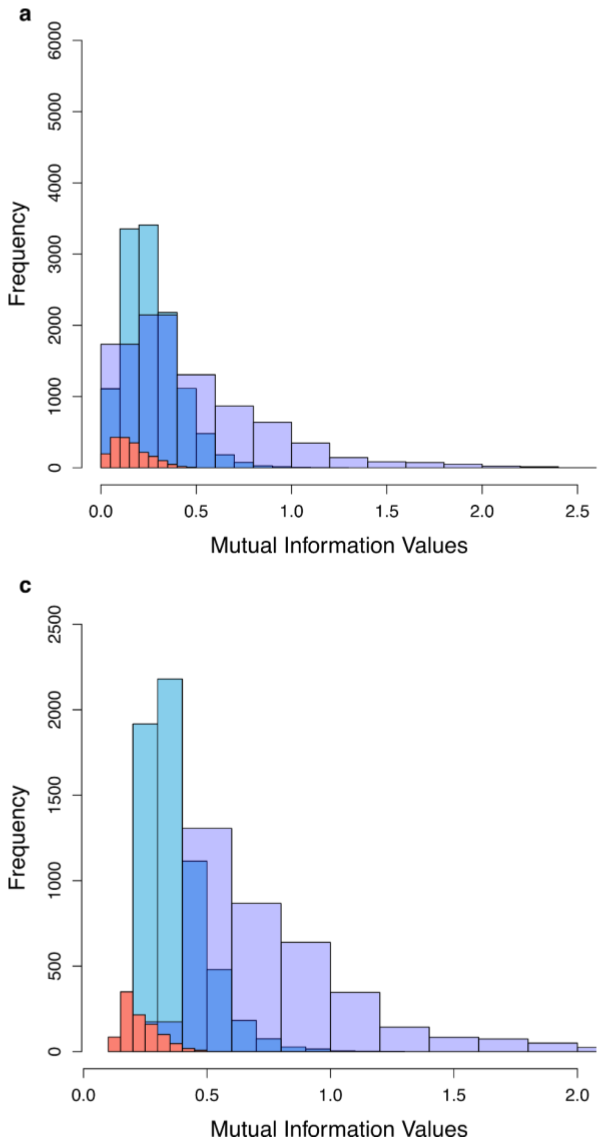

Figure S5: Mutual information distribution of the edges of the AIN and the UIN before and after thresholding. Related to STAR Methods. (**a**) The MI distribution of edges in the AIN before thresholding: Median= 0.24, Mean= 0.26 for TE-TE edge types, Median= 0.14, Mean= 0.156 for TE-IR edge types, and Median= 0.38, Mean= 0.49 for IR-IR edge types. (**b**) The MI distribution in the UIN before thresholding: Median= 0.15, Mean= 0.18 for TE-TE edge types, Median= 0.09, Mean= 0.10 for TE-IR edge types, and Median= 0.39, Mean= 0.50 for IR-IR edge type. (**c**) The MI distribution in the AIN after thresholding: Median= 0.34, Mean= 0.37 for TE-TE edge types, Median= 0.21, Mean= 0.23 for TE-IR edge types, and Median= 0.68, Mean= 0.77 for IR-IR edge types. (**d**) The MI distribution in the UIN after thresholding: Median= 0.31, Mean= 0.35 for TE-TE edge types, Median= 0.17, Mean= 0.19 for TE-IR edge types, and Median= 0.71, Mean= 0.81 for IR-IR edge types.

Methods S1: Details regarding data preprocessing and correction, network inference and its robustness against sample-size heterogeneity, and network post-processing

Cell type deconvolution

Gene and transcript-level reads were filtered for unwanted sequences using Cutadapt [1] v2.10, we also removed zero-length reads and retained only those with a count ≥ 10 in at least 95% of samples, resulting in 9777 genes and 11427 transcripts. Before correcting for batch effects, we calculated the cell type decomposition values using the Granulator v1.2.0 [2] package on the raw gene counts of the 9777 genes. We used the leukocyte gene signature matrix LM22 [3] as a reference matrix and applied the dtangle algorithm from Granulator resulting in 22 cell types: B cells memory, B cells naive, Dendritic cells activated, Dendritic cells resting, Eosinophils, Macrophages M0, Macrophages M1, Macrophages M2, Mast cells activated, Mast cells resting, Monocytes, Neutrophils, NK cells activated, NK cells resting, Plasma cells, T cells CD4 memory activated, T cells CD4 memory resting, T cells CD4 naive, T cells CD8, T cells follicular helper, T cells gamma delta, T cells regulatory (Tregs). Principal components (PCs) of the cell type proportions for the 336 individuals (107 unaffected and 229 affected) were calculated, and the first 5 PCs covering 56% of the variance were included for the batch correction process.

Batch correction

To account for confounding effects, we first corrected the gene and transcript-level data for the sequencing run using the `removeBatchEffect` function from the limma R package v3.50.1 [4,5]. The sequencing run constituted a huge effect, which can be seen in a PC analysis in Figure S2a. We then performed Surrogate Variable Analysis (SVA) [6] and identified 21 additional hidden batch effects. The decision on which variables to include for batch correction was carried out in two steps: 1) A canonical correlation analysis (CCA) which identified correlations between the known biological and technical covariates: sex, age, BMI, the 5 PCs of cell type proportions, GC content, total read pairs, study, status, RNA integrity number (RIN), plates, preparation batch, and medication information, since 48% of affected individuals were under some medication (see Table1 in the manuscript). The consumed medications were split into six categories: 'antidepressants', 'mood stabilisers', 'neuroleptica', 'tranquilizer', 'MAO inhibitors', and 'any medication'. We also included the 21 SVs, and the first 10 PCs of the gene counts after correcting for sequencing run (Figure S2b). 2) An association analysis using ANOVA of the aforementioned technical and biological covariates, 5 SVs extracted after correcting for sequencing run, the first 5 PCs of cell type decomposition values, the six categories of medication as predictors, and the first 10 PCs of the gene counts after correcting for sequencing run as the target variables (Figure S2c). The CCA identified high correlations between the calculated SVs and the PCs of gene expression values (corrected for sequencing run), where e.g., SV1 was highly correlated with PC1 (r=0.99), and SV2 with PC2 (r=0.97), indicating that the SVs effectively capture the variation represented by the PCs of gene expression data. The CCA also showed a high correlation between the GC content and PC1/SV1 (r=0.79), and a moderate correlation between the total read pairs and PC2/SV2 (r=0.46). It also identified a high correlation between the second principal component of cell type decomposition values (cellType_pc_2) with the study (r=0.68) and a moderate correlation with the total read pairs (r=0.45). The results also indicated moderate correlations between 'any medication' and 'antidepressants' with the status (r=0.46, r=0.44, respectively) and low correlation with

PC3/SV3 (r=0.36, r=0.34, respectively). The results from the ANOVA tests (Figure S2c) provided a clearer view of the contribution of the different variables to the variation of gene expression data captured by the PCs. It revealed a significant effect of 'any medication' and 'antidepressants' only on the third PC that explains a limited portion of the overall variance (less than 8%), see Table S16. Based on the above exploratory analyses, the final set of variables we chose to correct for included GC content, total read pair, and the top 5 PCs of cell type proportions, which even though they showed limited associations with the variation of the expression data, they reflect the cell type heterogeneity in blood samples, especially the imbalance between PBMCs used for affected individuals and the mix of PBMCs and whole blood samples for unaffected individuals. We disregard RIN to avoid overcorrection since it showed low correlation with the PCs. Moreover, although not shown to be significant contributors to variance in the data, we nonetheless correct for sex, age, and BMI as those variables have been shown in previous studies to be important confounders. We removed genes and transcripts that had negative values due to the subtraction of the modeled batch effects with the `removeBatchEffect` function, resulting in 7394 genes and 7334 transcripts.

ARACNE for network inference

For both the affected individuals (AIN) and unaffected individuals' networks (UIN), we used ARACNE (Algorithm for the Reconstruction of Accurate Cellular Networks) [7], an information-theoretic-based method designed for the reverse engineering of regulatory networks. Specifically, ARACNE builds a network in two steps. In the first step, the mutual information between each pair of input values is calculated and treated as an edge weight. In the second step, the majority of false-positive interactions are removed by applying the Data Processing Inequality (DPI) [8], where an edge with the smallest MI value in all network triplets is removed and regarded as an indirect interaction. For example, the weakest edge in a triplet (i,j,k), say (i,k), is removed if $MI(X_{ik}) \leq min(MI(X_{ij}), MI(X_{jk}))$.

Sample size effect analysis

In an initial analysis, our samples comprised affected and unaffected individuals only from the OPTIMA and BeCOME cohorts, with a total number of 210 affected and 63 unaffected individuals after outlier filtering. This huge sample size difference affects the network inference step, leading to uncertain conclusions regarding differences in the networks' structures. Figure S4 shows a PCA of the embeddings of the AIN (n=210), the UIN (n=63), as well as 10 different sub-AINs constructed by randomly sampling 60 samples, and 10 sub-AINs constructed by randomly sampling 150 samples from the affected group. These embeddings were calculated using the Graph2Vec technique [9], with an embedding vector dimension of 128. The results show the effect of sample size on the inferred networks' structures, which can be seen at the embedding level and is captured by PC1. Specifically, sub-AINs constructed with a higher sample size (150) are closer in the embedding space to the affected individuals' network constructed using the entire available samples (210) compared to the sub-AINs that are built using a smaller sample size (60). Therefore, to guarantee a high-quality network inference, unbiased by sample size effects, and enabling fair comparison of the two networks, we increased the sample size of the unaffected group by integrating the IST study, adding 37 control individuals. We would like to highlight that this analysis investigating the effect of sample size on network inference using graph embedding approaches constitute a first-of-its-kind effort that has not been explored before in the literature.

Network inference for affected and unaffected individuals

We infer the AIN for the affected group (n=210) and the UIN for the unaffected group (n=95) using both total gene expression (n=7394) and isoform ratios (n=7097) as input for ARACNE. Following the work of [10], we filter edges connecting features of the same gene to reduce bias in the networks and increase interpretability. This means we remove edges of type IR-IR connecting two isoforms of the same gene, and remove edges of type TE-IR connecting a gene with its isoform. The resulting AIN consists of 14,300 nodes and 21,324 edges, whereas the UIN consists of 14,447 nodes and 40,676 edges. This difference in the number of inferred edges between the two networks is due to the heterogeneity of the sample size since the UIN is built using a smaller sample size. This causes the UIN to have a higher number of edges with lower MI values compared to the AIN (Figure S5). Hence, to ensure a fair comparison of the two networks, we need to balance the number of edges and ensure that only edges with high MI values are included in the final networks. To this end, we filter out edges with low MI values in both networks (Figure S5a,b). For each edge type, we choose one threshold as the edge type-specific median MI value in the AIN, since it is more robustly constructed with a higher number of samples, i.e., 0.24 for TE-TE edge type, 0.14 for TE-IR edge type, and 0.38 for IR-IR edge type. We use this filtering threshold for both networks to obtain the final filtered networks (Figure S5c,d). This thresholding leads to the two networks having similar numbers in terms of #nodes, #edges, and #edges per edge type (Table 2), guaranteeing a fair comparison between the two networks.

Supplementary References

1. Martin, M. Cutadapt removes adapter sequences from high-throughput sequencing reads. *EMBnet J.* **17**, 10 (2011).

2. Pfister, Kuettel, et al. *Granulator*. (Bioconductor, 2021). doi:10.18129/B9.BIOC.GRANULATOR.
3. Newman, A. M. *et al.* Robust enumeration of cell subsets from tissue expression profiles. *Nat. Methods* **12**, 453–457 (2015).
4. Phipson, B., Lee, S., Majewski, I. J., Alexander, W. S. & Smyth, G. K. ROBUST HYPERPARAMETER ESTIMATION PROTECTS AGAINST HYPERVARIABLE GENES AND IMPROVES POWER TO DETECT DIFFERENTIAL EXPRESSION. *Ann. Appl. Stat.* **10**, 946–963 (2016).
5. Ritchie, M. E. *et al.* limma powers differential expression analyses for RNA-sequencing and microarray studies. *Nucleic Acids Res.* **43**, e47 (2015).
6. Leek, J. T., Johnson, W. E., Parker, H. S., Jaffe, A. E. & Storey, J. D. The sva package for removing batch effects and other unwanted variation in high-throughput experiments. *Bioinformatics* **28**, 882–883 (2012).
7. Margolin, A. A. *et al.* ARACNE: an algorithm for the reconstruction of gene regulatory networks in a mammalian cellular context. *BMC Bioinformatics* **7 Suppl 1**, S7 (2006).
8. Cover, T. M. & Thomas, J. A. *Elements of Information Theory*. (Wiley-Interscience, 1991).
9. Narayanan, A. *et al.* Graph2vec: Learning distributed representations of graphs. (2017) doi:10.48550/ARXIV.1707.05005.
10. Saha, A. *et al.* Co-expression networks reveal the tissue-specific regulation of transcription and splicing. *Genome Res* **27**, 1843–1858 (2017).
